# Efficacy of Leukocyte and Platelet-Rich Fibrin (L-PRF) as an adjunct to Open Flap Debridement (OFD) in periodontal intrabony defects: a systematic review and meta-analysis

**DOI:** 10.1186/s12903-026-09163-z

**Published:** 2026-07-09

**Authors:** Majid Shahmoradi, MohammadSoroush Sehat, Pedram Iranmanesh, Jaber Yaghini, Ahmad Mogharehabed, Mahdi Shahmoradi

**Affiliations:** 1 Department of Periodontics, School of Dentistry, Isfahan University of Medical Sciences, Isfahan, Iran; 2 Dental Implant Research Center, Dental Research Institute, School of Dentistry, Isfahan University of Medical Sciences, Isfahan, Iran; 3 Department of Endodontics, Dental Research Center, Dental Research Institute, School of Dentistry, Isfahan University of Medical Sciences, Isfahan, Iran; 4 Department of Periodontics, Dental Implant Research Center, Dental Research Institute, School of Dentistry, Isfahan University of Medical Sciences, Isfahan, Iran; 5 School of Dentistry, The University of Sydney, Sydney, Australia

**Keywords:** L-PRF, Intrabony defects, Periodontal regeneration, Autologous platelet concentrates, Meta-analysis

## Abstract

**Background:**

Leukocyte and platelet-rich fibrin (L-PRF) is an autologous biomaterial used in periodontal regenerative therapy due to its scaffold properties and sustained release of growth factors. This study aimed to evaluate the efficacy of L-PRF as an adjunct to open flap debridement (OFD) in the treatment of periodontal intrabony defects.

**Methods:**

In accordance with PRISMA guidelines, a literature search of PubMed, Scopus, Web of Science and Cochrane CENTRAL was conducted up to November 2025 by two independent reviewers for randomized controlled trials (RCTs) evaluating L-PRF as an adjunct to OFD. Primary outcomes assessed were changes in probing depth (PD) and clinical attachment level (CAL); secondary outcomes included changes in radiographic bone fill (RBF) and gingival margin level (GML). Risk of bias and certainty of evidence were assessed using Cochrane RoB 2 and the GRADE approach, respectively. Pooled mean differences (MD) and 95% confidence intervals (CI) were calculated. Exploratory subgroup analyses evaluated lesion type, application protocols and centrifugation settings.

**Results:**

Twenty-three RCTs were included. Twelve studies had low risk of bias, eight had some concerns and three had high risk. At 9 months (16 studies), L-PRF + OFD significantly improved PD (MD: 1.11 mm; 95% CI: 0.84–1.38) and CAL (MD: 1.13 mm; 95% CI: 0.79–1.48) compared with OFD alone (*p* < 0.05). Secondary outcomes (RBF and GML) also showed significant improvements. The certainty of evidence ranged from very low to moderate. Subgroup analyses suggested that defect morphology, application approach and centrifugation parameters may influence outcomes; however, these findings are exploratory and based on limited data.

**Conclusions:**

Adjunctive L-PRF appears to provide additional clinical and radiographic benefits in the management of periodontal intrabony defects when used as an adjunct to OFD. However, overall confidence in these findings is limited by very low to moderate certainty of evidence and substantial heterogeneity. Although outcomes may be influenced by defect characteristics and preparation protocols, current evidence does not support definitive clinical recommendations. Further well-designed, standardized multicenter RCTs with longer follow-up are required.

**Trial registration:**

PROSPERO registration number CRD42020168521.

**Supplementary Information:**

The online version contains supplementary material available at https://doi.org/10.1186/s12903-026-09163-z.

## Background

Leukocyte- and platelet-rich fibrin (L-PRF) is a second-generation autologous platelet concentrate (APC) derived from the patient’s blood [1]. It contains leukocytes with potential anti-inflammatory and anti-infective properties, while activated platelets release growth factors such as platelet-derived growth factor (PDGF), transforming growth factor-beta (TGF-β) and vascular endothelial growth factor (VEGF), which promote cell proliferation, angiogenesis and extracellular matrix formation [2, 3].

L-PRF can be used as a sole filling material or applied in different physical forms, as a clot, a compressed membrane, or in combinations of these forms, with or without other regenerative materials [4]. When applied to intrabony defects, L-PRF acts as a biological scaffold that promotes migration, proliferation, and differentiation of periodontal ligament cells and osteoblasts, thereby facilitating periodontal tissue regeneration [5, 6].

Although previous studies have generally reported favorable outcomes with adjunctive L-PRF, considerable heterogeneity in study design, defect characteristics and preparation protocols continue to limit definitive conclusions regarding its clinical efficacy compared with OFD alone. A systematic review by Castro et al. [7] evaluated the regenerative potential of L-PRF across multiple clinical applications. Moreover, a meta-analysis by Panda et al. [8], including four studies with a 9-month follow-up, reported statistically significant improvements in probing depth (PD) reduction, clinical attachment level (CAL) gain and radiographic bone fill (RBF).

Subsequently, a systematic review by Miron et al. [2], comprising 14 studies, reported improvements in PD, CAL and RBF. More recently, an updated analysis by Miron et al. [9], including 55 randomized controlled trials, has also indicated beneficial effects of APCs, including L-PRF, across various clinical indications, including periodontal regenerative procedures. However, this evidence also encompasses different treatment modalities and combination approaches with adjunctive regenerative biomaterials. Consequently, the magnitude and consistency of these effects remain uncertain due to substantial inter-study variability, limiting the ability to clearly isolate the independent adjunctive effect of L-PRF when used specifically with OFD in intrabony defects.

Given this background, the present study aims to conduct a systematic review and meta-analysis of randomized controlled trials (RCTs) to compare the clinical and radiographic outcomes of OFD combined with L-PRF versus OFD alone in treating intrabony defects. This study specifically evaluates changes in CAL, PD, RBF, and gingival margin level (GML). Furthermore, this review provides an updated synthesis with exploratory subgroup analyses based on follow-up duration, lesion type, treatment protocol, L-PRF preparation setting, and study design. These analyses may provide clinically relevant insights into the regenerative potential of L-PRF and will contribute to evidence-based recommendations for managing intrabony defects. The null hypothesis of this study is that the adjunctive use of L-PRF combined with OFD does not result in statistically significant differences in clinical and radiographic outcomes compared to OFD alone in the treatment of periodontal intrabony defects. 

## Methods

### Protocol, registration and focused question

This systematic review and meta-analysis is conducted according to the Cochrane Handbook for Systematic Reviews of Interventions [10] and is reported following the Preferred Reporting Items for Systematic Reviews and Meta-Analyses (PRISMA) checklists [11](Supplementary file S1). The protocol for this study was registered on PROSPERO with the registration number CRD42020168521. The focused question is, “Does using L-PRF improve clinical parameters (CAL, PD, GML) and RBF compared to using open flap debridement alone?”

### Eligibility criteria

The eligibility criteria were established based on the PICOS (Population, Intervention, Comparison, Outcomes, Study design) framework. To ensure the reproducibility of the study selection process, specific inclusion and exclusion criteria were explicitly defined as follows:

#### Inclusion criteria

- Population/Patient Characteristics (P): Systemically healthy adult patients (≥ 18 years) diagnosed with periodontitis, presenting with at least one periodontal intrabony defect.
- Intervention (I): OFD combined with the adjunctive use of L-PRF.
- Comparison (C): OFD alone.
- Outcomes (O): Studies reporting clear quantitative data (mean and standard deviation values at baseline and follow-up) for at least one of the primary clinical or radiographic parameters, including PD, CAL, GML or RBF.
- Study Design (S): RCTs, including both parallel-group and split-mouth designs, with a minimum follow-up duration of 6 months post-surgery.

#### Exclusion criteria

- Patient Characteristics: Patients with systemic diseases or conditions known to affect periodontal healing (e.g., diabetes mellitus), smokers and pregnant or lactating women.
- Intervention Variations: The use of other APCs (e.g., PRP, A-PRF) instead of standard L-PRF, the combination of L-PRF with other regenerative biomaterials (e.g., bone grafts or non-L-PRF barrier membranes) unless a distinct L-PRF + OFD arm was evaluated or L-PRF applications in other dental and medical fields.
- Defect Characteristics: Studies evaluating non-intrabony defects, such as furcation involvements or strictly suprabony defects.
- Outcome Reporting: Studies lacking sufficient quantitative data for meta-analysis or those from which data could not be extracted or obtained after contacting the authors.
- Study Design: Observational studies, non-randomized trials, case series, case reports, reviews and in vivo animal or in vitro studies.

### Study selection and data collection process

A systematic search of four electronic databases including PubMed/MEDLINE, Web of Science, Scopus and the Cochrane Central Register of Controlled Trials (CENTRAL) was conducted up to November 2025 without restrictions. Two independent reviewers (M.S. and MS.S.) performed the search in consultation with a clinical librarian. The references of included studies were also screened. All identified records were retrieved and managed using Mendeley (version 1.19). Detailed search strategies for each database are provided in Supplementary File S2.

After deleting duplicate records, the titles and abstracts of retrieval records were screened. Then, the full texts of potential records were scrutinized for eligibility. Two reviewers (MS and MSS) independently conducted all the screening assessments. Discrepancies were resolved through discussion with the third reviewer (JY). Additionally, data extraction was performed independently by the two reviewers (MS and MSS) and cross-verified, encompassing study characteristics, patient demographics, intrabony defect features and outcomes. Discrepancies were addressed through discussions with the third reviewer (JY). In the event of missing data, two emails were sent to the corresponding author over two weeks.

### Risk of bias assessment

Two reviewers (MS and MSS) independently evaluated the risk of bias according to the revised Cochrane risk-of-bias tool for randomized trials, RoB 2 [10, 12]. Studies were categorized by their risk of bias, with discrepancies resolved by consulting the third reviewer (JY).

### Outcome measures

Primary outcomes were defined as changes in PD and CAL, while secondary outcomes included RBF and GML changes. Clinical parameters (PD, CAL and GML) were measured utilizing periodontal probes. RBF was defined as the linear radiographic change in osseous defect depth, measured in millimeters from the alveolar crest to the base of the defect. Percentage bone fill values and CBCT-derived outcomes were not pooled. Where reported, measurements were based on standardized periapical radiographs using computer-assisted calibration or radiographic grids; however, calibration procedures and measurement error were not uniformly reported and could not be quantitatively adjusted.

### Statistical analysis

All meta-analyses were performed using Stata 17. Mean differences (MDs) and 95% confidence intervals (CIs) were calculated for continuous outcomes. Split-mouth and parallel-group trials were pooled using the reported summary statistics, including group means, standard deviations and sample sizes. Because paired standard deviations and within-participant correlation coefficients were not consistently reported for split-mouth trials, correlation-adjusted analyses could not be performed. This limitation was considered when interpreting the precision and certainty of the pooled estimates.

A random-effects model was applied to account for anticipated heterogeneity, assessed via Cochran’s Q and the I² index (I²>50% indicates substantial heterogeneity) [13]. To explore potential sources of heterogeneity, pre-specified subgroup analyses were conducted (lesion type, treatment protocol, L-PRF settings and study design); however, these were considered exploratory due to the limited number of included studies. Sensitivity analyses excluded trials with “high” or “some concerns” risk of bias.

Publication bias was assessed using funnel plots, Egger’s and Begg’s tests, but interpreted cautiously given their low power with fewer than 10 studies. Certainty of evidence was evaluated using the Grading of Recommendations Assessment, Development and Evaluation (GRADE) approach [14]. Statistical significance was set at *p* < 0.05.

## Results

### Study selection

**Fig. 1:**
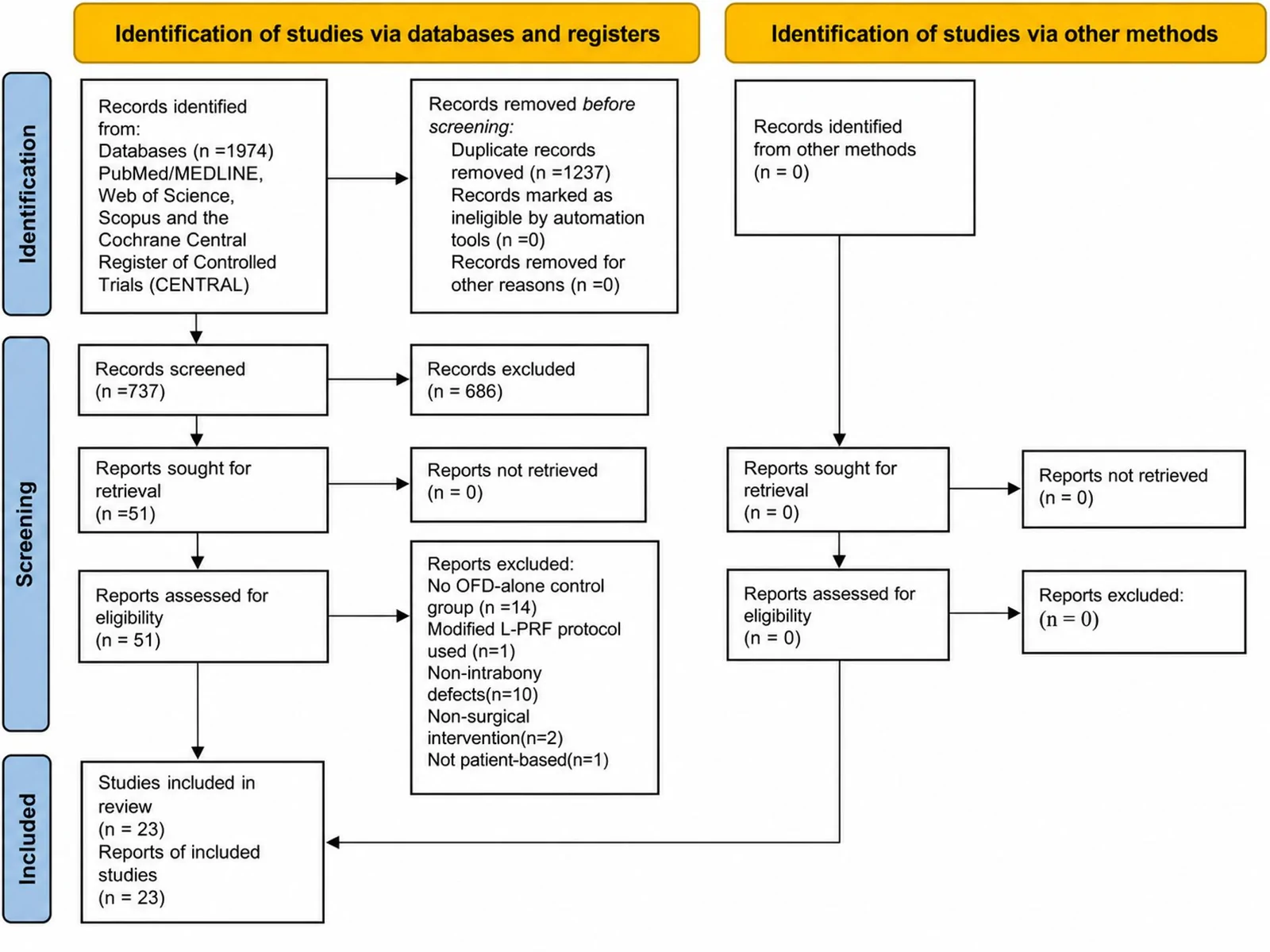
PRISMA 2020 flow diagram for study selection

A systematic literature search identified 1974 potential articles, with no additional records obtained from alternative sources. After removing duplicates, 737 titles and abstracts underwent initial screening. Subsequently, 686 articles were excluded based on the relevance of the exclusion criteria, leaving 51 for full-text review. After retrieving all records, 28 were excluded for various reasons (Supplementary file S3). Ultimately, 23 studies met the inclusion criteria for qualitative synthesis. The meta-analysis encompassed all studies meeting the inclusion criteria except one with no reported standard deviation [15]. The PRISMA flow diagram detailing the study selection process is presented in Fig. 1.

### Study characteristics

The 23 RCTs encompassing the review are characterized in Table 1. The geographic distribution of the trials included 18 [15–32] from India and 2 studies from Turkey [33, 34], with the remainder conducted in Egypt [35], Vietnam [36] and Saudi Arabia [37], each representing one study. These trials included either parallel (*n* = 15) or split-mouth designs (*n* = 8), focusing on systemically healthy, non-smoking subjects with intrabony defects. Sample sizes ranged from 10 to 30 sites per group of the trials. Lesion specificity varied, with 10 trials addressing two- and/or three-wall defects and 13 trials exclusively investigating three-wall defects. All trials mandated initial periodontal therapy before surgical intervention, with a follow-up period spanning 6, 9 and 12 months. Centrifugation protocols to prepare L-PRF predominantly utilized a setting of 3000 rpm for 10 min, employed in 15 trials. Alternative centrifugation protocols were reported in the remaining trials, indicating a degree of procedural diversity (Table 1).

**Table 1:** Characteristics of included studies

Author, year	Country	Study Design	Sample Size	Lesion Type	Hardware	Setting	Follow-up period
(Sharma & Pradeep, 2011) [30]	India	Parallel	28	All 3 walls	C	3000 rpm for 10 m	9 months
(Thorat et al., 2011) [31]	India	Parallel	16	2 and 3 walls	A	3000 rpm for 12 m	9 months
(Pradeep et al., 2012) [27]	India	Parallel	30	All 3 walls	C	3000 rpm for 10 m	9 months
(Rosamma et al., 2012) [29]	India	Split mouth	15	All 3 walls	B	3000 rpm for 10 m	12 months
(Ajwani et al., 2015) [28]	India	Split mouth	20	2 and 3 walls	C	3000 rpm for 10 m	9 months
(Pradeep et al., 2015) [26]	India	Parallel	30	All 3 walls	C	3000 rpm for 10 m	9 months
(Chandradas et al., 2016) [25]	India	Parallel	12	2 and 3 walls	C	3000 rpm for 10 m	9 months
(Kanoriya et al., 2016) [24]	India	Parallel	24	All 3 walls	C	3000 rpm for 10 m	9 months
(Martande et al., 2016) [23]	India	Parallel	30	All 3 walls	C	3000 rpm for 10 m	9 months
(Pradeep et al., 2016) [22]	India	Parallel	30	2 and 3 walls	C	3000 rpm for 10 m	9 months
(Arabacı et al., 2017) [34]	Turkey	Split mouth	26	All 3 walls	NI	2800 rpm for 12 m	9 months
(Bajaj et al., 2017) [17]	India	Parallel	27	All 3 walls	C	3000 rpm for 10 m	9 months
(Chatterjee et al., 2017) [16]	India	Parallel	30	All 3 walls	NI	373.3 g for 10 m	6, 9 months
(Patel et al., 2017) [20]	India	Split mouth	13	All 3 walls	C	3000 rpm for 10 m	6, 9, 12 months
(Pradeep et al., 2017) [21]	India	Parallel	29	All 3 walls	C	3000 rpm for 10 m	9 months
(Thorat et al., 2017) [19]	India	Split mouth	15	2 and 3 walls	C	3000 rpm for 12 m	12 months
(Yajamanya et al., 2017) [18]	India	Parallel	30	2 and 3 walls	NI	3000 rpm for 10 m	6, 9 months
(Kizildağ et al., 2018) [33]	Turkey	Split mouth	16	All 3 walls	D	3000 rpm for 10 m	6 months
(Bahammam & Attia, 2021) [37]	Saudi Arabia	Parallel	15	2 and 3 walls	NI	3000 rpm for 10 m	6 months
(Pham, 2021) [36]	Vietnam	Split mouth	30	2 and 3 walls	D	2500 rpm for 15 m	6, 12 months
(Razi et al., 2021) [15]	India	Parallel	10	2 and 3 walls	NI	NI	9 months
(Mubarak et al., 2023) [35]	Egypt	Parallel	10	2 and 3 walls	E	2700 rpm/12m	6 months
(Pendyala et al., 2025) [32]	India	Split mouth	25	All 3 walls	NI	400 g for 12 m	6, 9, 12 months

A: Process protocol, Nice, France, B: KW-70, AlmicroTM Instruments, Ambala Cantt., Haryana, India., C: R-4C, REMI, Mumbai, India. D: angle centrifuge, Hettich EBA 200, Germany. E: Intraspin; IntraLockTM, Boca Raton, FL, USA

### Risk of bias

**Fig. 2:**
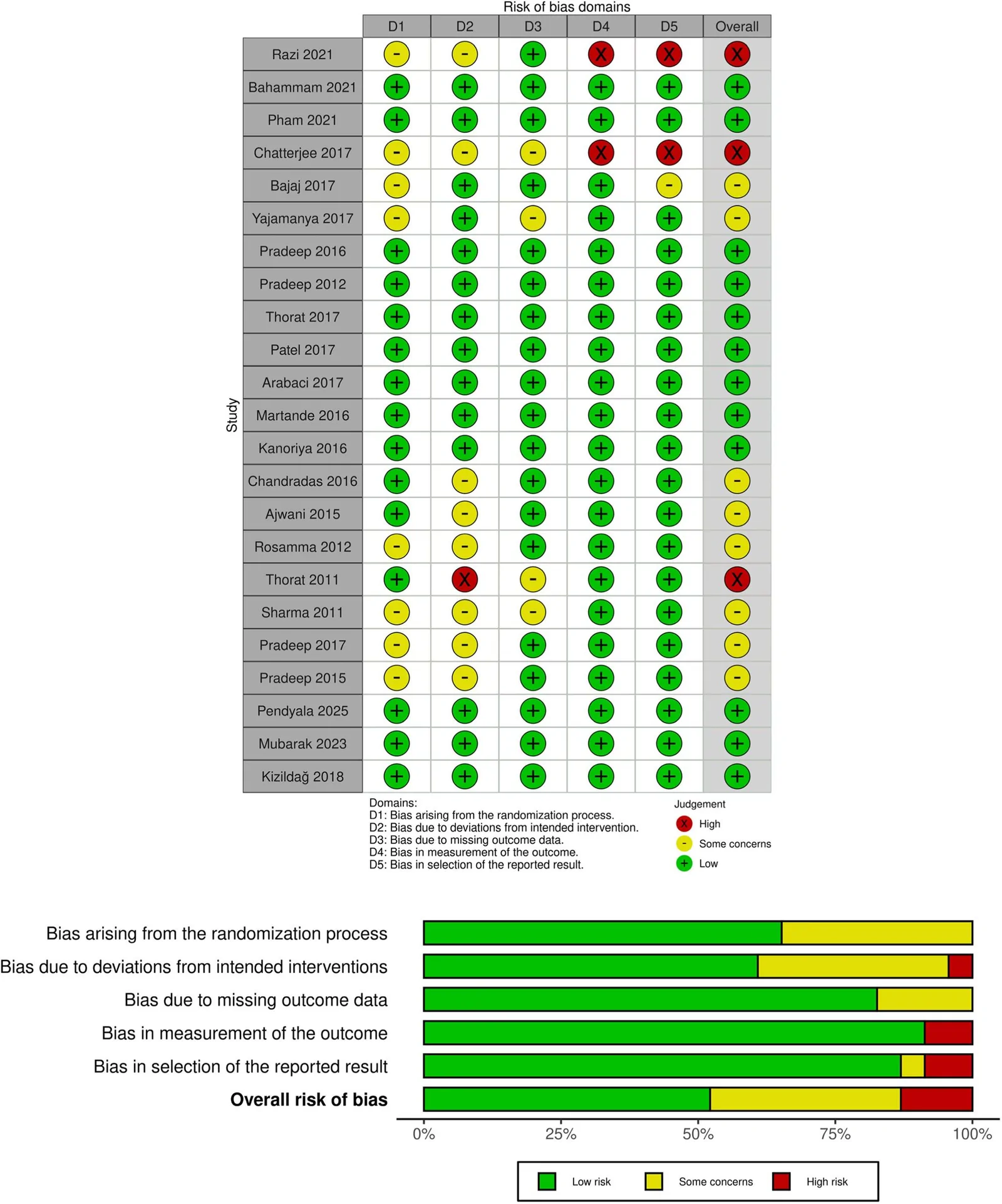
Risk of bias assessment of included randomized controlled trials using the RoB 2.0 tool. The figure displays the domain-level and overall risk of bias judgments for each included study, as well as a summary plot showing the proportion of studies rated as low risk, some concerns or high risk in each domain

Overall, 12 studies demonstrated a low risk of bias, 8 raised some concerns and 3 exhibited a high risk. High risk of bias was primarily associated with the measurement of outcomes, selection of reported results and deviations from intended interventions. Conversely, low risk of bias was observed in areas such as the randomization process and missing outcome data (Fig. 2).

### Primary outcomes

#### Probing Depth (PD)

**Fig. 3:**
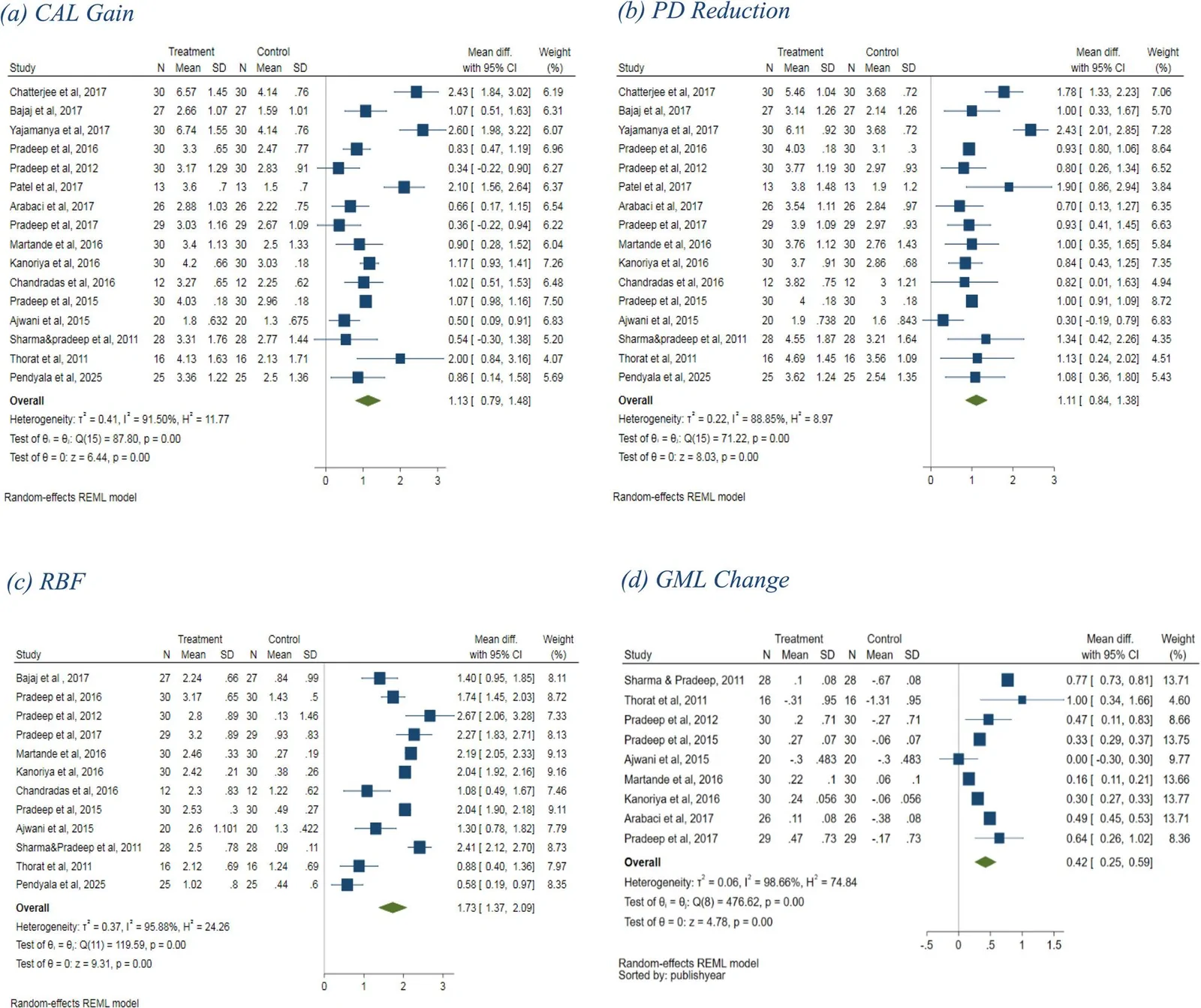
Meta-analysis of clinical outcomes at 9-month follow-up: **(a)** CAL gain, **(b)** PD reduction, **(c)** RBF and **(d)** GML change

The adjunctive use of L-PRF resulted in a statistically significant reduction in PD compared to OFD alone across all evaluated time points. At the 6-month follow-up (*n* = 7), the pooled MD was 1.42 mm (95% CI: 0.86 to 1.98; I^2^ = 78.15%). Independent pools of studies also demonstrated significant PD reductions at 9 months (*n* = 16, MD: 1.11 mm; 95% CI: 0.84 to 1.38; I^2^ = 88.85%) and at 12 months (*n* = 4, MD: 2.03 mm; 95% CI: 1.49 to 2.56; I^2^ = 75.35%) (Fig. 3a, Supplementary File S4).

#### Clinical Attachment Level (CAL)

Significant CAL gains in favor of the L-PRF group were consistently observed across different follow-up periods. At 6 months (*n* = 7), the pooled analysis showed an MD of 1.30 mm (95% CI: 0.59 to 2.01; I^2^ = 87.30%). Similarly, significant improvements were noted at 9 months (*n* = 16, MD: 1.13 mm; 95% CI: 0.79 to 1.48; I^2^ = 91.50%) and 12 months (*n* = 4, MD: 2.53 mm; 95% CI: 1.46 to 3.61; I^2^ = 92.53%) (Fig. 3b, Supplementary File S5).

### Secondary outcomes

#### Radiographic Bone Fill (RBF)

Radiographic evaluations indicated a statistically significant benefit of L-PRF in increasing RBF. The pooled MDs were 0.82 mm (95% CI: 0.33 to 1.31; I^2^ = 79.15%) for the 6-month evaluation (*n* = 4), 1.73 mm (95% CI: 1.37 to 2.09; I^2^ = 95.88%) for the 9-month evaluation (*n* = 12) and 1.63 mm (95% CI: 1.15 to 2.11; I^2^ = 43.95%) at 12 months (*n* = 3) (Fig. 3c, Supplementary File S6).

#### Gingival Margin Level (GML)

Changes in GML were statistically significant, favoring the L-PRF groups. At 9 months, nine studies evaluated this outcome, showing a pooled MD of 0.42 mm (95% CI: 0.25 to 0.59; I^2^ = 98.66%). A single study evaluating GML at 12 months reported a mean difference of 0.48 mm (Fig. 3d, Supplementary File S7).

### Subgroup analysis

#### Subgroup analysis by lesion type

**Fig. 4:**
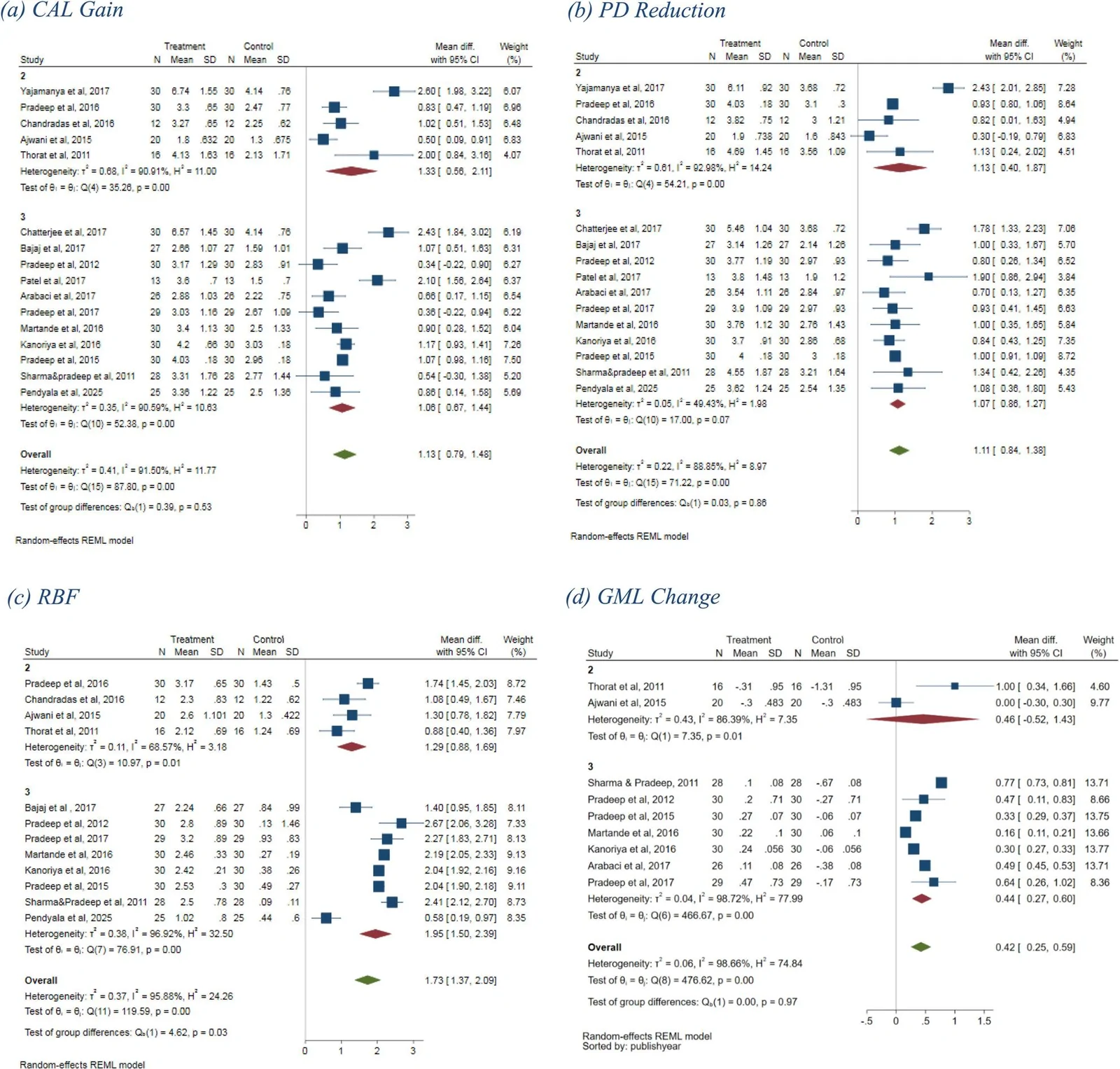
Subgroup analysis by defect morphology at 9-month follow-up: **(a)** CAL gain, **(b)** PD reduction, **(c)** RBF and **(d)** GML change. Note: Numbers 2 and 3 indicate the type of periodontal defect included in each subgroup—2 represents two-wall intrabony defects and 3 represents three-wall intrabony defects

At the 9-month follow-up, subgroup analyses assessed differences according to lesion morphology, comparing combined 2- and 3-wall defects (subgroup A) with strictly 3-wall defects (subgroup B). A statistically significant subgroup difference was observed only for RBF (*p* = 0.03), with strictly 3-wall defects showing a higher pooled RBF estimate (*n* = 8, MD: 1.95 mm; 95% CI: 1.50 to 2.39; I² = 96.92%) than combined defects (*n* = 4, MD: 1.29 mm; 95% CI: 0.88 to 1.69; I² = 68.57%). For the remaining clinical parameters (PD, CAL and GML), no significant inter-group differences were detected (Fig. 4).

#### Subgroup analysis by treatment protocol

At the 9-month follow-up, subgroup analyses evaluated five L-PRF application protocols (Protocol 1: one clot only; Protocol 2: one membrane only; Protocol 3: one clot and one membrane; Protocol 4: one clot and two membranes; Protocol 5: two membranes only). Statistically significant inter-group differences were observed exclusively for CAL gain (*p* = 0.03) and RBF (*p* < 0.01).

**Fig. 5:**
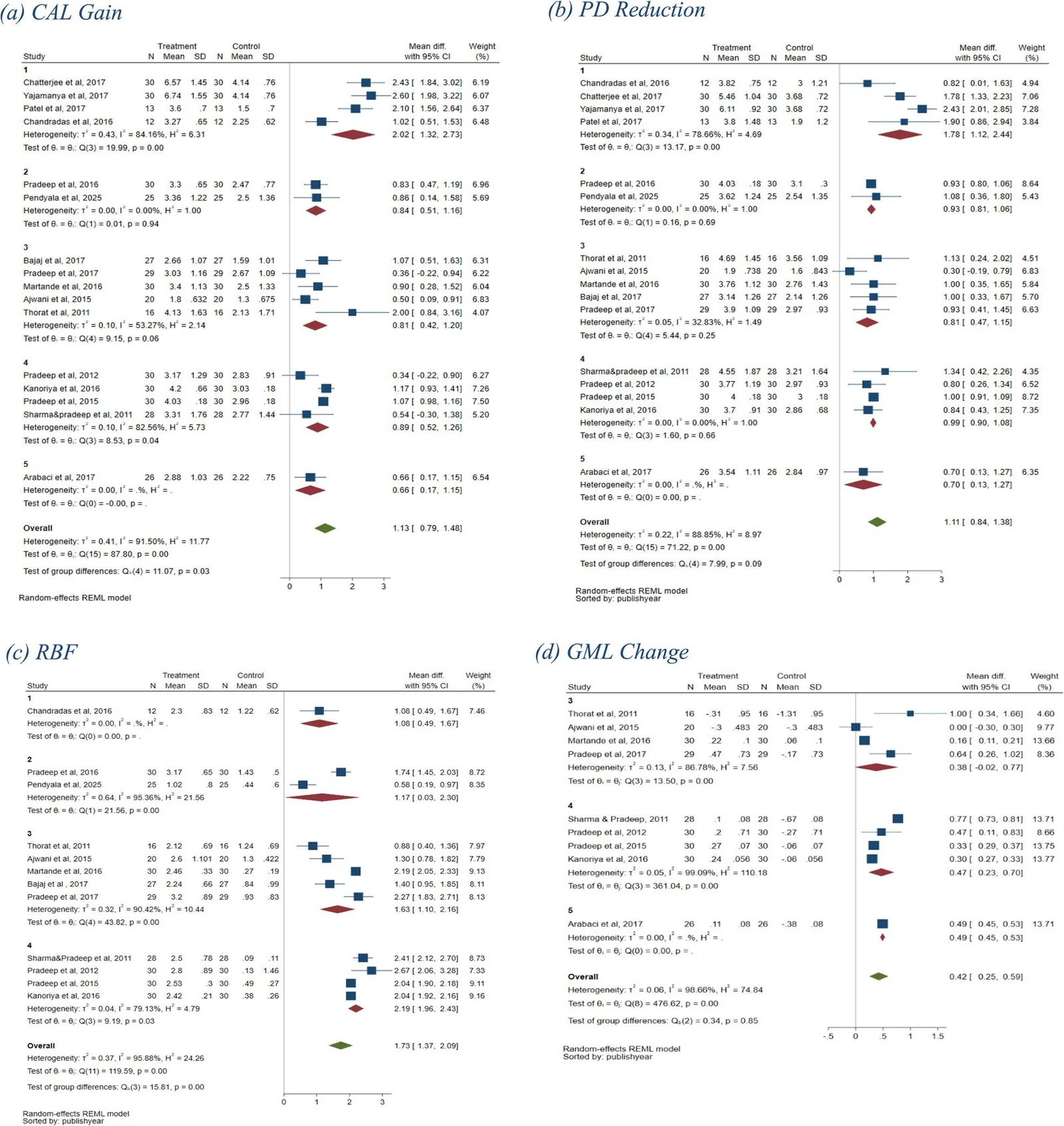
Subgroup analysis by treatment protocol at 9-month follow-up: **(a)** CAL gain, **(b)** PD reduction, **(c)** RBF and **(d)** GML change. Note: Numbers 1 to 5 refer to the treatment protocols used in each subgroup: 1 = One clot only , 2 = One membrane only, 3 = One clot and one membrane, 4 = One clot and two membranes, 5 = Two membranes only

In exploratory subgroup analyses, Protocol 1 showed the largest pooled CAL estimate (*n* = 4, MD: 2.02 mm; CI: 1.32 to 2.73; I^2^ = 84.16%). For RBF, Protocol 4 showed the largest pooled RBF estimate (*n* = 4, MD: 2.19 mm; 95% CI: 1.96 to 2.43; I^2^ = 79.13% ), whereas Protocol 3 and Protocol 1 showed pooled mean differences of 1.63 mm (*n* = 4, 95% CI: 1.10 to 2.16 ; I^2^ = 90.42% ) and 1.08 mm (*n* = 1, 95% CI: 0.49 to 1.67), respectively. No significant inter-group differences were detected for PD reduction (*p* = 0.09) and GML changes (*p* = 0.85) (Fig. 5).

#### Subgroup analysis by L-PRF preparation setting

**Fig. 6:**
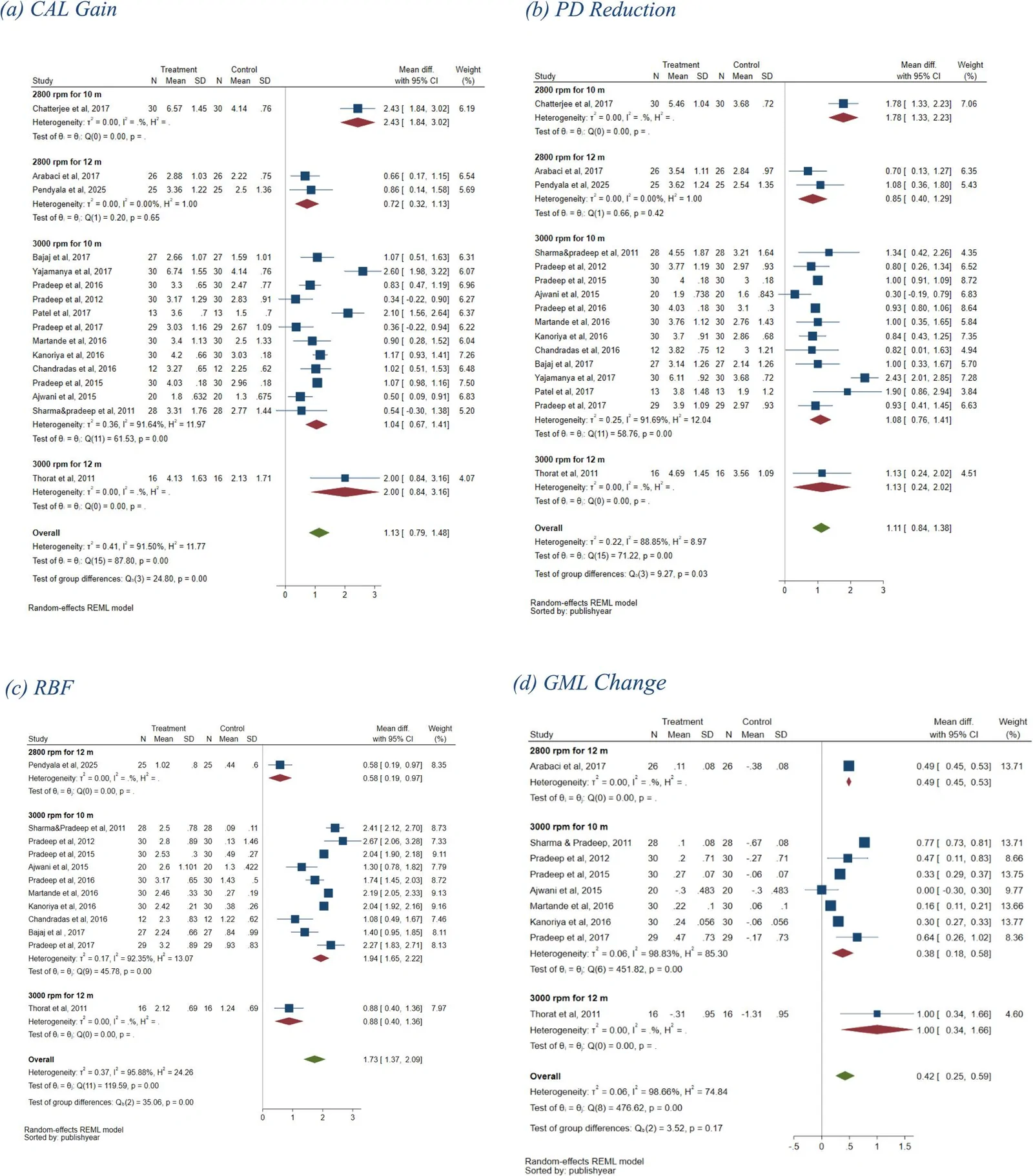
Subgroup analysis by L-PRF preparation setting at 9-month follow-up: **(a)** CAL gain, **(b)** PD reduction, **(c)** RBF and **(d)** GML change

Subgroup analyses at 9 months compared four L-PRF preparation protocols (2800 or 3000 rpm for 10–12 min). Because several preparation-setting subgroups were represented by a single trial, within-subgroup heterogeneity estimates were not applicable for those comparisons and the findings should be interpreted as exploratory rather than confirmatory. The available data suggested that preparation setting may influence PD and CAL outcomes, but the limited number of studies within some categories precludes definitive conclusions (Fig. 6).

Regarding RBF, the 3000 rpm/10 min setting was the most frequently reported protocol and yielded the largest pooled RBF estimate (*n* = 10, MD: 1.94 mm; 95% CI: 1.65 to 2.22), although heterogeneity remained substantial (I² = 92.35%).

Finally, preparation settings had no significant effect on GML changes (*p* = 0.17) (Fig. 6).

#### Subgroup analysis by study design

A subgroup analysis comparing split-mouth and parallel-group trials did not show statistically significant differences between study designs across the evaluated outcomes (Supplementary Files S4–S7).

### Sensitivity analyses and publication bias

Excluding studies with high or some concerns risk of bias did not substantially alter the pooled estimates for both primary and secondary outcomes (Supplementary File S8). Furthermore, funnel plots and statistical tests (Egger’s and Begg’s) indicated no major asymmetry across the evaluated outcomes (Supplementary File S9).

### Certainty of evidence

Based on the GRADE approach, the overall certainty ranged from very low to moderate (Summary of Findings Table 2). The evidence was primarily downgraded due to serious inconsistency arising from substantial heterogeneity, as well as imprecision and potential publication bias in some analyses. Risk of bias and indirectness were not considered serious concerns.

**Table 2:** Summary of findings

Efficacy of leukocyte and platelet-rich fibrin (L-PRF) as an Adjunct to Open Flap Debridement in Periodontal Intrabony Defects								
Outcome	Relative effect (MD, 95% CI)	Number of lesions (trials)	**Certainty of evidence**					Quality of evidence (GRADE)
			Risk of bias	Inconsistency	Indirectness	Imprecision	Publication bias	
CAL (6 month)	1.30 (0.59, 2.01)	288 lesions (7)	No concern	Serious concern	No concern	No concern	Serious concern	Low quality (⊕⊕OO)
CAL (9 month)	1.13(0.79, 1.48)	812 lesions (16)	No concern	Very serious concern	No concern	No concern	No concern	Low quality (⊕⊕OO)
CAL (12 month)	2.53 (1.46, 3.61)	146 lesions (4)	No concern	Very serious concern	No concern	Serious concern	Very serious concern	Very low quality (⊕OOO)
PD (6 month)	1.42(0.86, 1.98)	288 lesions (7)	No concern	Serious concern	No concern	No concern	No concern	Moderate quality ⊕⊕⊕O
PD (9 month)	1.11(0.84, 1.38)	812 lesions (16)	No concern	Serious concern	No concern	No concern	No concern	Moderate quality (⊕⊕⊕O)
PD (12 month)	2.03(1.49, 2.56)	146 lesions (4)	No concern	Serious concern	No concern	Serious concern	No concern	Low quality (⊕⊕OO)
GML (9 month)	0.42(0.25, 0.59)	478 lesions (9)	No concern	Very serious concern	No concern	No concern	No concern	Low quality (⊕⊕OO)
RBF (6 month)	0.82(0.33, 1.31)	160 lesions (4)	No concern	Serious concern	No concern	No concern	No concern	Moderate quality (⊕⊕⊕O)
RBF (9 month)	1.73(1.37, 2.09)	614 lesions (12)	No concern	Very serious concern	No concern	No concern	No concern	Low quality (⊕⊕OO)
RBF (12 month)	1.63(1.15, 2.11)	120 lesions (3)	No concern	No concern	No concern	Serious concern	No concern	Moderate quality (⊕⊕⊕O)

Summary of evidence from randomized controlled trials evaluating the efficacy of leukocyte and platelet‑rich fibrin (L‑PRF) as an adjunct to open flap debridement (OFD) in the treatment of periodontal intrabony defects. The table reports relative effects for clinical outcomes at various follow‑up periods, number of included lesions/trials and certainty of evidence based on GRADE domains (risk of bias, inconsistency, indirectness, imprecision and publication bias)

Patients: Systemically healthy adults (≥ 18 years) diagnosed with periodontal disease presenting intrabony periodontal defects

Setting: Clinical periodontal treatment settings in randomized controlled trials (RCTs) evaluating surgical management of intrabony defects

Intervention: Open flap debridement (OFD) combined with leukocyte- and platelet-rich fibrin (L-PRF)

Comparison: Open flap debridement (OFD) alone without the use of L-PRF

Specifically, the evidence for PD reduction and RBF change was rated as moderate to low certainty. The certainty for CAL ranged from low to very low across all follow-up periods, while the evidence for GML change was rated as low at the 9-month evaluation.

### Safety and adverse events

The reporting of safety outcomes was inconsistent across the included trials. Of the 23 studies, 15 explicitly described uneventful healing with no significant adverse events reported [15–27, 30, 31]. In the remaining eight studies, postoperative complications were not specifically reported. Among the studies that provided safety data, L-PRF was well tolerated, and no withdrawals related to adverse events were observed.

## Discussion

### Summary of principal findings

This systematic review and meta-analysis evaluated the adjunctive efficacy of L-PRF combined with OFD for the treatment of periodontal intrabony defects. Compared with OFD alone, adjunctive L-PRF was associated with significant improvements in PD reduction, CAL gain, GML and RBF change across 6-, 9- and 12-month follow-up periods. Notably, the largest effect estimates were observed at 12 months, suggesting that the regenerative benefits of L-PRF may be maintained over time.

However, these findings should be interpreted with caution due to the substantial heterogeneity observed across the included studies. It is also important to highlight the limitations of the subgroup analyses. Evaluations of centrifugation protocols, application methods and defect morphology were restricted by a small pool of available studies, in some instances, relying on a single trial. Because no adjustments for multiple comparisons were performed, these specific findings are inherently exploratory and serve primarily to generate hypotheses for future research.

Safety data were inconsistently reported across the included studies. In RCTs that reported such outcomes, no significant adverse events were reported. 

### Comparison with existing literature and biological rationale

The findings of the present meta-analysis are in agreement with previous systematic reviews, which have generally reported beneficial effects of L-PRF on clinical and radiographic outcomes in periodontal intrabony defects [7, 9, 38]. In some clinical settings, L-PRF has been used in combination with bone grafts or compared with barrier membranes in the management of complex defects, supporting its role as a versatile adjunctive biomaterial [39]. In contrast, the present analysis specifically focuses on the adjunctive use of L-PRF with OFD alone, thereby providing a more targeted assessment of its clinical contribution in a controlled surgical context.

From a biological perspective, the observed clinical improvements align with the well-established properties of L-PRF. As originally described by Choukroun et al. [1], L-PRF is a second-generation platelet concentrate prepared without anticoagulants, resulting in a dense fibrin matrix that functions as a temporary biological scaffold. This matrix facilitates early wound healing through sustained release of growth factors and cytokines involved in cellular migration, angiogenesis, and tissue regeneration [5]. In addition, clinical and experimental evidence has demonstrated that platelet-rich fibrin matrices can modulate inflammatory responses and enhance both soft and hard tissue healing, providing a plausible mechanistic explanation for the observed improvements in periodontal regenerative parameters [38, 40]. 

### Clinical relevance and implications of exploratory subgroup analyses

Adjunctive L-PRF demonstrated statistically significant benefits over OFD alone, with a mean additional PD reduction of 1.11 mm and CAL gain of 1.13 mm. While these differences may appear modest in absolute terms, they are consistent with the effect sizes reported for established regenerative approaches. In this regard, a comprehensive meta-analysis by Nibali et al. [41] reported mean additional improvements of 1.20 mm in PD reduction and 1.34 mm in CAL gain for regenerative procedures compared with OFD alone.

From a clinical interpretation perspective, periodontal regenerative outcomes should not be evaluated solely on isolated parameters. As proposed by Trombelli et al. [42], treatment success is more appropriately conceptualized using composite outcome measures that integrate attachment gain and pocket closure. Within this framework, the adjunctive effect observed with L-PRF may be considered potentially clinically relevant when it contributes to improved pocket resolution and periodontal maintainability, although direct assessment against composite success thresholds was not possible in the present analysis.

Beyond the overall treatment effects, the exploratory subgroup analyses provided preliminary but clinically informative insights into potential sources of variability in L-PRF outcomes. With respect to defect morphology, the more pronounced RBF observed in studies restricted to 3-wall defects is biologically plausible, as contained defects may provide a more favorable environment for clot stabilization, space maintenance and bone regeneration [43]. Variations in centrifugation protocols and application modalities also showed different trends in clinical and radiographic outcomes, which may reflect the sensitivity of L-PRF to preparation parameters, fibrin architecture, clot handling and growth factor release kinetics [44, 45]. However, given the study-level nature of these analyses, the limited number of studies in several subgroups and the lack of adjustment for multiple comparisons, these findings should be interpreted as exploratory rather than confirmatory and should not be used alone to define an optimal L-PRF protocol or application method.

From a practical perspective, the clinical implementation of L-PRF requires consideration of procedural feasibility. Unlike off-the-shelf biomaterials, its preparation involves chairside blood collection and immediate centrifugation, making the final product sensitive to operator technique and protocol standardization [44]. Variations in handling or delays before processing may influence the structural and biological characteristics of the fibrin matrix [45]. Although L-PRF offers advantages such as autologous origin and low material cost, these procedural requirements may affect its routine clinical applicability in some settings. 

### Limitations

Several limitations of this systematic review and meta-analysis should be acknowledged.

First, the included studies primarily relied on surrogate clinical and radiographic outcomes, such as PD, CAL, and RBF, which were generally reported as isolated parameters. Although these measures are widely accepted indicators of periodontal treatment response, their independent assessment may not fully capture overall therapeutic success. As proposed by Trombelli et al. [42], the composite outcome measure (COM), which integrates attachment gain with pocket resolution, may provide a more clinically meaningful evaluation of regenerative outcomes. However, insufficient reporting of raw outcome data across the included studies precluded COM-based analyses in the present review.

Second, none of the included trials provided histological confirmation of true periodontal regeneration. Consequently, it remains unclear whether the observed clinical and radiographic improvements reflected true periodontal regeneration, including formation of new cementum, periodontal ligament and alveolar bone, or healing by repair mechanisms such as long junctional epithelium formation.

Third, long-term outcome data were limited. Most studies evaluated outcomes over relatively short follow-up durations, typically ranging from 6 to 12 months, and robust evidence regarding long-term tooth survival and stability of clinical improvements remains lacking. In addition, patient-reported outcome measures (PROMs), recurrence of disease activity and sustained bleeding on probing control were inconsistently assessed. This limits the evaluation of both the long-term durability of treatment effects and their clinical relevance from a patient-centered perspective.

Furthermore, considerable clinical and methodological heterogeneity was observed across the included studies, including variations in defect morphology, centrifugation protocols, surgical techniques and L-PRF application modalities. Split-mouth and parallel-group trials were also pooled without adjustment for intra-individual correlation because paired standard deviations and within-participant correlation coefficients were not reported in the primary studies. Although subgroup analysis by study design did not show significant differences between designs, the lack of correlation-adjusted estimates remains a methodological limitation and may have affected variance estimates and study weights.

The exploratory subgroup analyses were also associated with important limitations, including small sample sizes within several subgroups, substantial between-study heterogeneity and the absence of adjustment for multiple comparisons. Accordingly, these findings should be interpreted as hypothesis-generating rather than confirmatory.

Moreover, the current evidence base demonstrates substantial geographic and authorship clustering. Approximately 78% of the included trials were conducted in India, with several studies originating from overlapping research groups. This concentration may limit the external validity and generalizability of the findings, as similarities in clinical protocols and operator expertise could have influenced treatment outcomes.

Finally, the overall certainty of evidence ranged from very low to moderate according to the GRADE framework, primarily due to inconsistency related to substantial heterogeneity, with imprecision and potential publication bias affecting some analyses. Consequently, confidence in the estimated effect sizes remains limited.

Future randomized controlled trials should prioritize standardized L-PRF preparation and reporting, incorporate composite outcomes and PROMs, and include longer follow-up periods.

## Conclusion

In conclusion, adjunctive L-PRF appears to provide additional clinical and radiographic benefits in the management of periodontal intrabony defects when used alongside OFD. However, these findings are based on evidence of very low to moderate certainty and should be interpreted with caution, given the substantial heterogeneity across studies. Exploratory analyses suggest that defect characteristics, treatment protocols, and preparation parameters may influence outcomes, although the available data do not allow firm conclusions. Further well-designed, adequately powered randomized controlled trials with standardized methodologies and longer follow-up are required to clarify the clinical relevance and long-term stability of these effects. 

## Supplementary Information

Supplementary Material 1.

Supplementary Material 2.

Supplementary Material 3.

Supplementary Material 4.

Supplementary Material 5.

Supplementary Material 6.

Supplementary Material 7.

Supplementary Material 8.

Supplementary Material 9.

## Data Availability

Data are available from the corresponding author upon reasonable request.

## Abbreviations

L-PRF: Leukocyte and platelet-rich fibrin
OFD: Open flap debridement
APCs: Autologous platelet concentrates
PD: Probing depth
CAL: Clinical attachment level
RBF: Radiographic bone fill
GML: Gingival margin level
RCTs: Randomized controlled trials
PDGF: Platelet-derived growth factor
TGF-β: Transforming growth factor-beta
VEGF: Vascular endothelial growth factor
PRF: Platelet-rich fibrin
PRISMA: Preferred reporting items for systematic reviews and meta-analyses
MD: Mean difference
CI: Confidence interval
COM: Composite outcome measure
PROMs: Patient-reported outcome measures
