# Supplementary material for: Efficacy of Leukocyte and Platelet-Rich Fibrin (L-PRF) as an adjunct to Open Flap Debridement (OFD) in periodontal intrabony defects: a systematic review and meta-analysis
Source: BMC Oral Health. 2026 Jul 9;26:1917. doi: 10.1186/s12903-026-09163-z (PMC13637309; doi:10.1186/s12903-026-09163-z)
Supplement: Supplementary file 2 [file 12903_2026_9163_MOESM2_ESM.docx]

| **Database** | **Search Strategy** | **Number of retrieval records** |
| --- | --- | --- |
| PubMed/ Medline | ((alveolar bone loss[MeSH Terms]) OR (periodontal) OR (periodontitis[MeSH Terms]) OR (bony defects) OR (intrabony) OR (Intra-bony) OR (infrabony) OR (infra-bony) OR (intraosseous) OR (infraosseous) OR (endosseous) OR (osseous)) AND ((Platelet-Rich Fibrin[MeSH Terms]) OR (Leukocyte and Platelet Rich Fibrin[MeSH Terms]) OR (L-PRF) OR (PRF)) AND (randomized clinical trial) | 285 |
| Scopus | TITLE-ABS-KEY ( ( ( alveolar AND bone AND loss ) OR ( periodontal ) OR ( periodontitis ) OR ( bony AND defects ) OR ( intrabony ) OR ( intra-bony ) OR ( infrabony ) OR ( infra-bony ) OR ( intraosseous ) OR ( infraosseous ) OR ( endosseous ) OR ( osseous ) ) AND ( ( platelet-rich AND fibrin ) OR ( leukocyte AND platelet AND rich AND fibrin ) OR ( l-prf ) OR ( prf ) ) AND ( ( randomized AND clinical AND trial ) OR ( randomized AND controlled AND trial ) OR ( clinical AND trial ) ) ) | 404 |
| Web of Science | ALL=(((alveolar bone loss) OR (periodontal) OR (periodontitis) OR (bony defects) OR (intrabony) OR (Intra-bony) OR (infrabony) OR (infra-bony) OR (intraosseous) OR (intraosseous) OR (endosseous) OR (osseous)) AND ((Platelet-Rich Fibrin) OR (Platelet Rich Fibrin) OR (Leukocyte and Platelet Rich Fibrin) OR (L-PRF) OR (PRF)) AND ( (randomized clinical trial) OR (randomized controlled trial) OR (RCT))) | 263 |
| Cochrane Central | ( (alveolar bone loss) OR (periodontal) OR (periodontitis) OR (bony defects) OR (intrabony) OR (Intra-bony) OR (infrabony) OR (infra-bony) OR (intraosseous) OR (intraosseous) OR (endosseous) OR (osseous)) AND ((Platelet-Rich Fibrin) OR (Platelet Rich Fibrin) OR (Leukocyte and Platelet Rich Fibrin) OR (L-PRF) OR (PRF) ) | 1022 |
