# Supplementary material for: Efficacy of Leukocyte and Platelet-Rich Fibrin (L-PRF) as an adjunct to Open Flap Debridement (OFD) in periodontal intrabony defects: a systematic review and meta-analysis
Source: BMC Oral Health. 2026 Jul 9;26:1917. doi: 10.1186/s12903-026-09163-z (PMC13637309; doi:10.1186/s12903-026-09163-z)
Supplement: Supplementary file 3 [file 12903_2026_9163_MOESM3_ESM.docx]

**Excluded Studies from the Systematic Review**

| No. | Study | Journal | Year | Reason for Exclusion |
| --- | --- | --- | --- | --- |
| 1 | Chadwick JK, Mills MP, Mealey BL. Clinical and Radiographic Evaluation of Demineralized Freeze-Dried Bone Allograft Versus Platelet-Rich Fibrin for the Treatment of Periodontal Intrabony Defects in Humans | J Periodontol | 2016 | Not having OFD alone as a control group. |
| 2 | Gupta SJ, Jhingran R, Gupta V, Bains VK, Madan R, Rizvi I. Efficacy of platelet-rich fibrin vs. enamel matrix derivative in the treatment of periodontal intrabony defects: a clinical and cone beam computed tomography study | J Int Acad Periodontol | 2014 | Not having OFD alone as a control group. |
| 3 | Aydemir Turkal H, Demirer S, Dolgun A, Keceli HG. Evaluation of the adjunctive effect of platelet-rich fibrin to enamel matrix derivative in the treatment of intrabony defects. Six-month results of a randomized, split-mouth, controlled clinical study | J Clin Periodontol | 2016 | Not having OFD alone as a control group. |
| 4 | Lekovic V, Milinkovic I, Aleksic Z, Jankovic S, Stankovic P, Kenney EB, et al. Platelet-rich fibrin and bovine porous bone mineral vs. platelet-rich fibrin in the treatment of intrabony periodontal defects | J Periodontal Res | 2012 | Not having OFD alone as a control group. |
| 5 | Agarwal A, Gupta ND, Jain A. Platelet rich fibrin combined with decalcified freeze-dried bone allograft for the treatment of human intrabony periodontal defects: a randomized split mouth clinical trail | Acta Odontol Scand | 2016 | Not having OFD alone as a control group. |
| 6 | Joshi AA, Padhye AM, Gupta HS. Platelet derived growth factor-BB levels in gingival crevicular fluid of localized intrabony defect sites treated with platelet rich fibrin membrane or collagen membrane containing recombinant human platelet derived growth factor-BB: A randomized clinical trial | J Periodontol | 2019 | Not having OFD alone as a control group. |
| 7 | Csifó-Nagy BK, Sólyom E, Bognár VL, Nevelits A, Dőri F. Efficacy of a new-generation platelet-rich fibrin in the treatment of periodontal intrabony defects: a randomized clinical trial | BMC Oral Health | 2021 | Modified version of L-PRF |
| 8 | Naqvi A, Gopalakrishnan D, Bhasin MT, Sharma N, Haider K, Martande S. Comparative Evaluation of Bioactive Glass Putty and Platelet Rich Fibrin in the Treatment of Human Periodontal Intrabony Defects: A Randomized Control Trial | J Clin Diagn Res | 2017 | Not having OFD alone as a control group. |
| 9 | Basireddy A, Prathypaty SK, Yendluri DB, Potharaju SP. Demineralized freeze-dried bone allograft with or without platelet-rich fibrin in the treatment of mandibular Degree II furcation defects: A clinical and cone beam computed tomography study | J Indian Soc Periodontol | 2019 | Not having OFD alone as a control group. |
| 10 | Elgendy EA, Abo Shady TE. Clinical and radiographic evaluation of nanocrystalline hydroxyapatite with or without platelet-rich fibrin membrane in the treatment of periodontal intrabony defects | J Indian Soc Periodontol | 2015 | Not having OFD alone as a control group. |
| 11 | Bansal C, Bharti V. Evaluation of efficacy of autologous platelet-rich fibrin with demineralized-freeze dried bone allograft in the treatment of periodontal intrabony defects | J Indian Soc Periodontol | 2013 | Not having OFD alone as a control group. |
| 12 | Shah M, Patel J, Dave D, Shah S. Comparative evaluation of platelet-rich fibrin with demineralized freeze-dried bone allograft in periodontal infrabony defects: A randomized controlled clinical study | J Indian Soc Periodontol | 2015 | Not having OFD alone as a control group. |
| 13 | Elbehwashy MT, Hosny MM, Elfana A, Nawar A, Fawzy El-Sayed K. Clinical and radiographic effects of ascorbic acid-augmented platelet-rich fibrin versus platelet-rich fibrin alone in intra-osseous defects of stage-III periodontitis patients: a randomized controlled clinical trial | Clin Oral Investig | 2021 | Not having OFD alone as a control group. |
| 14 | Bodhare GH, Kolte AP, Kolte RA, Shirke PY. Clinical and radiographic evaluation and comparison of bioactive bone alloplast morsels when used alone and in combination with platelet-rich fibrin in the treatment of periodontal intrabony defects-A randomized controlled trial | J Periodontol | 2019 | Not having OFD alone as a control group. |
| 15 | Treatment of Unfavorable Intrabony Defects with Autogenous Bone Graft in Combination with Leukocyte- and Platelet-Rich Fibrin or Collagen Membranes: A Non-Inferiority Study | DENTISTRY JOURNAL | 2024 | Not Intra-Bony Defects |
| 16 | Comprehensive analysis of bone tissue in extraction sockets of third molars after leukocyte and platelet rich fibrin and photobiomodulation applications | Clin Oral Investig | 2024 | Not Intra-Bony Defects |
| 17 | Impact of L-PRF on pain and healing outcomes in lower third molar surgery: a randomized split-mouth trial | Braz Oral Res | 2024 | Not Intra-Bony Defects |
| 18 | Comparison of histologic and radiographic changes of sockets grafted with LPRF and sockets without intervention after tooth extraction | Oral and Maxillofacial Surgery | 2024 | Not Intra-Bony Defects |
| 19 | Comparison of the effect of A-PRF and L-PRF application to palatal donor sites on quality of life and wound healing after free gingival graft surgery | Quintessence International | 2024 | Not Intra-Bony Defects |
| 20 | Evaluation of leukocyte-platelet rich fibrin as an antibiotic slow-release biological device in the treatment of moderate periodontitis: a randomized controlled clinical trial | BMC ORAL HEALTH | 2024 | Not Intra-Bony Defects |
| 21 | Peri-implant mucosal enhancement using leukocyte platelet rich fibrin under Sohn's poncho technique: A randomized controlled clinical trial | Clinical Advances in Periodontics | 2024 | Not Intra-Bony Defects |
| 22 | Treatment of multiple adjacent gingival recessions using leucocyte- and platelet-rich fibrin with coronally advanced flap: a 12-month split-mouth controlled randomized clinical trial | CLINICAL ORAL INVESTIGATIONS | 2024 | Not Intra-Bony Defects |
| 23 | Vertical Guided Bone Regeneration: Leukocyte and PRF Bone-Block vs A Mixture of Autogenous Bone with DBBM. A Split-Mouth Randomized Controlled Trial with Follow-up Up to 1 Year After Implant Loading | Int J Periodontics Restorative Dent | 2025 | Not Intra-Bony Defects |
| 24 | Horizontal Guided Bone Regeneration: L-PRF Bone Block Versus a Mixture of Autogenous Bone with Deproteinized Bovine Bone Mineral-A Split-Mouth RCT Study with a 25-Month Follow-up | International Journal of Oral and Maxillofacial Implants | 2025 | Not Intra-Bony Defects |
| 25 | Evaluation of leukocyte-platelet rich fibrin as an antibiotic slow-release biological device in the treatment of moderate periodontitis: a randomized controlled clinical trial | BMC ORAL HEALTH | 2024 | Not Surgical |
| 26 | Comparative evaluation of L-PRF and ofloxacin incorporated L-PRF as an adjunctive to SRP-A split-mouth randomized clinical trial | JOURNAL OF PHARMACY AND BIOALLIED SCIENCES | 2024 | Not Surgical |
| 27 | Instructions for the use of L-PRF in different clinical indications | Periodontology 2000 | 2025 | Not Patient Based |
| 28 | Treatment of Unfavorable Intrabony Defects with Autogenous Bone Graft in Combination with Leukocyte- and Platelet-Rich Fibrin or Collagen Membranes: A Non-Inferiority Study | Medicina (Kaunas) | 2024 | Not having OFD alone as a control group. |
