# Supplementary material for: Efficacy of Leukocyte and Platelet-Rich Fibrin (L-PRF) as an adjunct to Open Flap Debridement (OFD) in periodontal intrabony defects: a systematic review and meta-analysis
Source: BMC Oral Health. 2026 Jul 9;26:1917. doi: 10.1186/s12903-026-09163-z (PMC13637309; doi:10.1186/s12903-026-09163-z)
Supplement: Supplementary file 5 [file 12903_2026_9163_MOESM5_ESM.docx]

**CAL Analyses**

# Section 1: 6-Month Follow-Up:

## Table: Overall Forest Plot

## Table: Subgroup – Clot

## Table: Subgroup – Hardware

## Table: Subgroup – Lesion Type

## Table: Subgroup – Setting

## Table: Subgroup – Study Design

# Section 1: 9-Month Follow-Up:

## Table: Overall Forest Plot

## Table: Subgroup – Clot

## Table: Subgroup – Hardware

## Table: Subgroup – Lesion Type

## Table: Subgroup – Setting

## Table: Subgroup – Study Design

# Section 1: 12-Month Follow-Up:

## Table: Overall Forest Plot

## Table: Subgroup – Clot

## Table: Subgroup – Hardware

## Table: Subgroup – Lesion Type

## Table: Subgroup – Setting

## Table: Subgroup – Study Design
