# Supplementary material for: Efficacy of Leukocyte and Platelet-Rich Fibrin (L-PRF) as an adjunct to Open Flap Debridement (OFD) in periodontal intrabony defects: a systematic review and meta-analysis
Source: BMC Oral Health. 2026 Jul 9;26:1917. doi: 10.1186/s12903-026-09163-z (PMC13637309; doi:10.1186/s12903-026-09163-z)
Supplement: Supplementary file 8 [file 12903_2026_9163_MOESM8_ESM.docx]

**Sensitivity Analysis**

# PD Analysis :

## 6-Month Follow-Up

## 9-Month Follow-Up

## 12-Month Follow-Up

# CAL Analysis :

## 6-Month Follow-Up

## 9-Month Follow-Up

## 12-Month Follow-Up

# IBD Analysis :

## 6-Month Follow-Up

## 9-Month Follow-Up

## 12-Month Follow-Up

# GML Analysis :

## 9-Month Follow-Up
