# Supplementary material for: Efficacy of Leukocyte and Platelet-Rich Fibrin (L-PRF) as an adjunct to Open Flap Debridement (OFD) in periodontal intrabony defects: a systematic review and meta-analysis
Source: BMC Oral Health. 2026 Jul 9;26:1917. doi: 10.1186/s12903-026-09163-z (PMC13637309; doi:10.1186/s12903-026-09163-z)
Supplement: Supplementary file 9 [file 12903_2026_9163_MOESM9_ESM.docx]

**Funnel Plots**

| Groups | Egger's reg | p | Kendall's Tau | p |
| --- | --- | --- | --- | --- |
| 6 PD | -2.23 | 0.2333 | -9.00 | 0.2296 |
| 9 PD | 0.45 | 0.6970 | 33.00 | >.99 |
| 12 PD | -0.96 | 0.7147 | -2.00 | 0.7341 |
| 6 CAL | -7.12 | 0.0122 | -9.00 | 0.2296 |
| 9 CAL | 1.36 | 0.4164 | 19.00 | 0.3731 |
| 12 CAL | 17.57 | 0.0001 | 4.00 | 0.3082 |
| 6 IBD | -2.54 | 0.4795 | -1.00 | >.99 |
| 9 IBD | -2.29 | 0.1940 | -23.00 | 0.0868 |
| 12 IBD | -1.34 | 0.4072 | -1.00 | >.99 |
| 9 GML | 0.88 | 0.3904 | 10.00 | 0.3481 |

***Table 1. Evaluating publication bias through Egger's regression test and Begg's rank correlation test***

# PD Funnel Plot

## 6-Month Follow-Up:

## 9-Month Follow-Up:

## 12-Month Follow-Up:

# CAL Funnel Plot

## 6-Month Follow-Up:

## 9-Month Follow-Up:

## 12-Month Follow-Up:

# IBD Funnel Plot

## 6-Month Follow-Up:

## 9-Month Follow-Up:

## 12-Month Follow-Up:

# GML Funnel Plot

## 9-Month Follow-Up:
